# The End of the Year

**Published:** 1892-12

**Authors:** 


### HOMŒOPATHIC PHYSICIAN,

A MONTHLY JOURNAL OF

HOMŒOPATHIC MATERIA MEDICA AND CLINICAL MEDICINE.

"If our school ever gives up the strict inductive method of Hahnemann, we are lost, and deserve only to be mentioned as a caricature in the history of medicine."—CONSTANTINE HERING.

Vol. XII. DECEMBER, 1892. No. 12.

### EDITORIAL.

THE END OF THE YEAR.—With the present number THE HOMŒOPATHIC PHYSICIAN ends the twelfth year of its existence. In all that time it has never swerved from the plain principles of Hahnemann. This assertion has been repeatedly made in these pages as each year draws to its close. But as new subscribers are continually coming forward to peruse its pages, for the benefit of such we repeat the statement.

Agreeably to our expectations, THE HOMŒOPATHIC PHYSICIAN has been cordially sustained by the profession during the past year. In evidence of this, to it has been confided the publication of the transactions of the International Hahnemannian Association. These are now appearing in its pages, and the profession can form an excellent idea of the exceedingly interesting character of the sessions, held at Narragansett Pier in June last.

For the good-will thus manifested the editor extends his warmest thanks, and announces the continuance of the journal during the new year on exactly the same principles as in the past.

Every subscriber should now come to the assistance of the editor by inducing some friend to subscribe, and by contributing articles for its pages that will instruct the beginner and confirm the faith of the wavering.

